# Chronic kidney disease enhances alternative pathway activity: a new paradigm

**DOI:** 10.1172/JCI188353

**Published:** 2025-05-01

**Authors:** Diana I. Jalal, Joshua M. Thurman, Richard J.H. Smith

**Affiliations:** 1 Division of Nephrology, Department of Medicine, University of Iowa Carver College of Medicine, Iowa City, Iowa, USA.; 2 Center for Access and Delivery Research and Evaluation, Iowa City VA Health Care System, Iowa City, Iowa, USA.; 3 Division of Renal Diseases and Hypertension, Department of Medicine, Anschutz Medical Campus, University of Colorado, Aurora, Colorado, USA.; 4 Molecular Otolaryngology and Renal Research Laboratories, University of Iowa, Iowa City, Iowa, USA.

## Abstract

Reduced kidney function is associated with increased risk of cardiovascular disease in addition to kidney disease progression. Kidney disease is considered an inflammatory state, based on elevated levels of C-reactive protein and inflammatory cytokines. A key mediator of cardiovascular and kidney disease progression in the setting of reduced kidney function is systemic and vascular inflammation. However, the exact pathways that link chronic kidney disease (CKD) with inflammation remain incompletely understood. For decades it has been known that factor D, the main activator of the alternative complement pathway, is increased in the plasma of patients with reduced kidney function. Recent biomarker evidence suggests alternative pathway activation in this setting. CKD, therefore, seems to alter the balance of alternative pathway proteins, promoting inflammation and potentially exacerbating complement-mediated diseases and CKD-associated complications. In this manuscript, we review the impact of reduced kidney function on biomarkers of the alternative complement pathway and the implications of alternative pathway activation on cardiovascular disease and kidney disease progression. Importantly, we highlight the need for ongoing research efforts that may lead to opportunities to target the alternative pathway of complement withx the goal of improving kidney and cardiovascular outcomes in persons with reduced kidney function.

## Introduction

Chronic kidney disease (CKD), one of the most common chronic diseases, is estimated to afflict 850 million people worldwide (1), including 35.5 million people in the United States (2). CKD is defined as reduced kidney function based on estimation of glomerular filtration rate (GFR) or evidence of kidney damage most commonly in the form of albuminuria (3). Both indicators of kidney disease are powerful predictors of adverse outcomes, including kidney disease progression and cardiovascular disease (CVD).

A wealth of evidence indicates that reduced GFR is, itself, an independent risk factor for kidney disease progression and for CVD (4–7). A recent analysis in the Japanese population with mild-moderate CKD that utilized a machine learning supervised model found that an estimated GFR of less than 50 mL/min/1.73 m^2^ is the most powerful predictor of kidney disease progression and the development of end-stage kidney disease (ESKD) (8). Furthermore, low kidney function at the time of biopsy is a powerful predictor of ESKD in several immune-mediated diseases including IgA nephropathy (9), pauci-immune necrotizing glomerulonephritis (10), and lupus nephritis (11). Reduced GFR is additionally a predictor of cardiovascular events and mortality, and there is a stepwise increase in the risk of CVD as the GFR declines. The risk of CVD is 11 times and more than 20 times higher in those with estimated GFR in the ranges of 30–44 and 15–29 mL/min/1.73m^2^, respectively, as compared with age-matched controls with normal kidney function (6).

That reduced GFR alone is an important predictor of adverse kidney and cardiovascular outcomes suggests that GFR-related factors (i.e., molecules that accumulate with reduced kidney function) may contribute to systemic and vascular inflammation in CKD. Considering the pivotal role vascular inflammation plays in kidney and CVD onset and progression, the identification of GFR-related mediators of vascular inflammation is key to developing targeted therapies to stem the progression of kidney disease and CVD in CKD.

## Vascular inflammation in CKD

**Figure 1:**
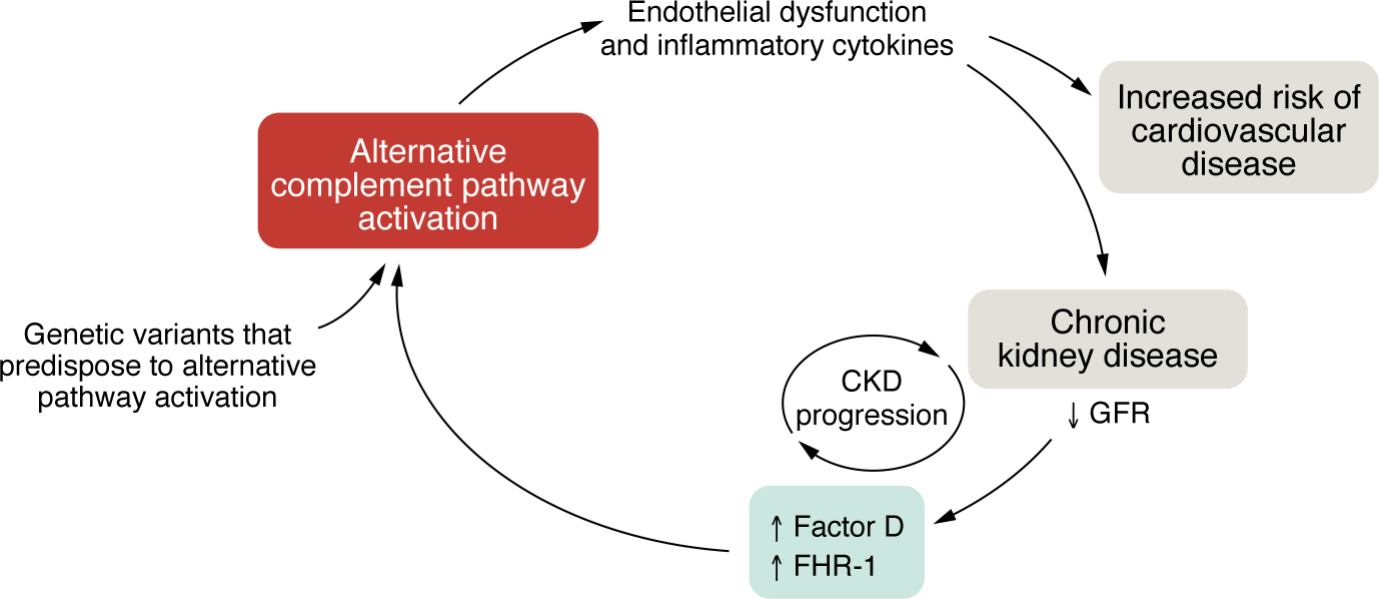
Conceptual model. Systemic and vascular inflammation contribute to CKD onset and progression. We propose that reduced kidney function contributes to alternative pathway lability in CKD and further fuels systemic and vascular inflammation, resulting in a cycle that not only aggravates the underlying kidney disease, but also increases the risk of CVD.

Vascular inflammation, the hallmark of atherosclerotic vascular disease, is characterized by endothelial dysfunction, chronic systemic and vascular wall inflammation, and oxidative stress (12). Nitric oxide (NO) protects the integrity of the endothelium by inhibiting inflammation and thrombosis (13). Notably, reduced NO is necessary for the development and progression of experimental kidney disease that mimics human disease (14, 15). As illustrated in Figure 1, endothelial dysfunction contributes not only to CVD but also to kidney disease onset and progression (16–18). Patients with CKD exhibit increased measures of endothelial dysfunction compared with non-CKD patients, and these, in turn, are prospective markers of CVD in CKD subjects (19–26).

A key mediator of vascular inflammation is the ubiquitous transcription factor NF-κB (27–30). NF-κB is activated by oxidative stress, and its activation upregulates proinflammatory genes including IL-1, IFN-γ, and TNF- α (31) and ultimately increases levels of IL-6 (32). Of note, systemic biomarkers of inflammation such as C-reactive protein and IL-6 are known to be increased and to associate with adverse kidney and cardiovascular outcomes in those with CKD (33, 34). More recently, several studies have identified protein members of the TNF receptor superfamily as predictors of incident CKD and kidney disease progression in the general population and in individuals with diabetes (35, 36). While greater degrees of oxidative stress are evident in CKD, drugs that target prooxidant pathways have not been shown to reduce the risk of kidney or CVD progression in CKD (37). This may be the result of the interrelatedness and complexity of the pro- and antioxidant pathways. However, it is also plausible that other more powerful proinflammatory pathways may be at play in CKD. In this instance, CKD-associated oxidative stress may represent a downstream effect of the activation of such pathways. While modern proteomics have led to several advances regarding the molecular pathways of inflammation and oxidative stress (35, 36, 38), the molecular links between CKD and systemic and vascular inflammation and oxidative stress remain incompletely understood.

Considering the pivotal role vascular inflammation plays in kidney and CVD onset and progression, the identification of GFR-related mediators of vascular inflammation is key to developing targeted therapies to stem the progression of kidney disease and CVD in CKD. Here, we review the known link between the alternative pathway and several complement-mediated kidney diseases, we evaluate the evidence that links reduced kidney function (including in nonimmune CKD) to higher levels of biomarkers of the alternative pathway, and we propose a potential role for the alternative complement pathway in kidney disease progression and in CVD in CKD (Figure 1).

## The alternative pathway in CVD and CKD

### The alternative pathway of complement

**Figure 2:**
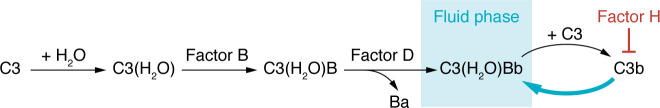
The alternative pathway of complement is in a perpetual state of activation and regulation in the fluid phase, a process termed “tickover”. The endothelium is exposed to the alternative pathway of complement and is susceptible to dysregulation of the pathway.

The complement cascade, an important component of innate immunity, is composed of 3 pathways including the classical, mannose-binding lectin, and alternative pathways and is controlled through a balance of activating and regulatory proteins (39). The alternative pathway comprises C3, factor B, factor D, and properdin. Factor D is produced as a proenzyme and requires cleavage by the enzyme MASP-3 to become functional (40). Several proteins restrict activation of the alternative pathway, including factor I, factor H, CD55, CD46, and CR1. Activation of the complement system serves as one of the first lines of defense in innate immunity and in the recognition and elimination of invading microorganisms (41). While the classical and mannose-binding lectin pathways are activated by antibodies and other pattern-recognizing molecules (42), the alternative pathway is constitutively activate through a self-amplifying process called C3 tick-over (Figure 2) (39).

The process of tick-over involves the continual generation of C3b, triggered by hydrolysis of C3, which leads to the generation of C3(H_2_O) (43). C3(H_2_O) forms a fluid-phase C3 convertase (C3[H_2_O]Bb) with factor B after its cleavage by factor D (44, 45). This fluid-phase C3 convertase is more resistant to inactivation factors H and I (both complement regulating proteins) than the solid-phase C3 convertase (C3bBb) (44–46), with formation of C3(H_2_O) also enhanced by certain surfaces (47). As C3a and C3b have downstream biologic effects, we consider cleavage of C3 by the convertases to represent activation of the system. Changes in transcription of complement proteins, on the other hand, do not necessarily indicate activation of the system per se, and generation of complement activation fragments should be demonstrated to conclude that activation has occurred.

Additional factors also influence the tendency towards activation in an individual. Polymorphisms in complement-activating genes and complement-regulating genes affect the overall degree of complement activity and in aggregate define “the complotype” (48). An individual’s age and sex can influence complement activity (49). Finally, local biochemical conditions, including pH or the presence of NH_3_, can also increase the tendency towards activation of the alternative pathway (50).

A healthy alternative pathway is essential for immune surveillance, removal of cellular debris, and organ regeneration (41). Control of this system involves a complex interplay among the alternative pathway protein levels, complement-regulating proteins, the complotype, and conditions within a tissue (41).

### The alternative pathway in kidney disease

Alternative pathway activation contributes to several kidney diseases (51). In some diseases (e.g., C3 glomerulopathy [C3G] and complement-mediated thrombotic microangiopathy/atypical hemolytic uremic syndrome [aHUS]), uncontrolled complement activation is the primary driver of kidney injury (52). In other kidney diseases, secondary activation of the alternative pathway occurs and contributes to disease progression. For example, there is strong preclinical and clinical evidence for a role of the alternative pathway in immune-complex membranoproliferative glomerulonephritis (53), lupus nephritis (54, 55), membranous nephropathy (56), IgA nephropathy (57), antineutrophil cytoplasmic antibody associated vasculitis (58), and acute tubular injury (59, 60). Although the underlying mechanisms of complement activation vary across these conditions, shared involvement of the complement system suggests that there are anatomic or physiologic features of the kidney that render it particularly susceptible to alternative pathway-mediated injury.

Many of the mechanisms of complement dysregulation first identified in C3G and aHUS have subsequently been observed in the other, more prevalent, kidney diseases. A good example is autoantibodies against factor H (FHAA), which were first appreciated as a cause of aHUS but have recently been detected in patients with C3G, recurrent membranous nephropathy, and monoclonal gammopathy of renal significance (61, 62). Similarly, the role of factor H–related proteins (FHRs) in complement control has been elucidated by studying C3G and then translated to other diseases. For example, elevated levels of FHR1 and FHR5 have been shown to promote alternative pathway activation and to correlate with faster disease progression in IgA nephropathy (63, 64). In diabetic kidney disease, alternative pathway activation and reduced expression of factor H have been reported (65, 66) and urinary levels of FHR2 have been shown to predict CKD progression (67). Hyperglycemia may cause acquired impairment of complement regulatory proteins (68). It is also possible that glomerular damage nonspecifically impairs complement regulation in the kidney (69). Although complement activation in diseases such as aHUS can trigger acute tissue inflammation, persistent activation can also lead to irreversible tissue damage and fibrosis. Several preclinical studies have suggested that chronic complement activation is an important driver of tubulointerstitial fibrosis in the kidney (70, 71). Thus, the pathophysiological role of the alternative pathway appears to span a much broader spectrum of diseases than initially thought.

### The alternative pathway in vascular disease

Because the alternative pathway is constitutively active via the tick-over mechanism, it is reasonable to envision that increased tick-over or an imbalance between complement activators and regulators could render the alternative pathway more labile (52, 72, 73). Considering that the endothelium is continually exposed to proteins of the alternative complement pathway, the endothelium may be particularly susceptible to a more labile complement system.

Increased complement activity may contribute to the high risk of CVD in CKD. There is evidence that the endothelium expresses *C3* and the complement factor B gene (*CFB*) (74, 75) and that this expression is upregulated by proinflammatory cytokines known to be increased in CKD, such as IL-6 and TNF-α (74, 76, 77). In addition, CVD is common in patients with an impaired ability to control activation of the alternative pathway (e.g., the acute phase of aHUS) (78). More recently, the expression of FHR1, which competitively reduces factor H function, has been found to associate with atherosclerotic CVD (79). These associations between complement activation and vascular disease are consistent with our observation that an elevation of factor B cleavage fragments correlates directly with albuminuria and inversely with brachial artery flow-mediated dilation, both indicators of endothelial dysfunction and independent predictors of CVD (20–26, 80).

One of the strongest links between *CFH* polymorphisms and disease is with age-related macular degeneration (AMD), where *CFH* polymorphisms are estimated to contribute to approximately 50% of AMD cases (81–85). Since CVD (particularly atherosclerotic disease) and AMD share pathogenic mechanisms (e.g., lipid deposition and thickening of connective tissue), some investigators have evaluated the potential role of *CFH* polymorphisms in CVD (86, 87). For example, the H402 allele of *CFH* is associated with an increased risk of AMD and also predicts future risk of myocardial infarction (88). Similarly, the *CFH* polymorphism p.Glu936Asp, which associates with lower factor H levels and predisposes to aHUS, predicts CVD and new onset albuminuria in patients with type 2 diabetes (89).

It is also worth noting that researchers have recently identified many intracellular functions for complement proteins. These noncanonical functions of complement proteins can directly affect endothelial cells (90) and macrophages within vascular plaques (91).

Collectively, these data have led us to hypothesize that activation of the alternative pathway may play a role in vascular inflammation, which in addition to contributing to CKD onset and progression, is magnified in the presence of reduced kidney function.

## Reduced kidney function alters alternative pathway protein expression

### Factor D

Factor D, an approximately 24 kD serine protease, is produced by the adipocytes and macrophages in adipose tissue and is the rate-limiting step in the activation of the alternative pathway of complement (92). It circulates in its active form, and when it encounters factor B complexed with either C3b or C3(H_2_O), it cleaves a lysine-arginine bond in factor B to make an active convertase (93–95). Most importantly, factor D is filtered by the kidney and its serum concentration is impacted by kidney function (96). Several studies have evaluated the levels of factor D in CKD and are summarized in Table 1. In 1985, Volanakis et al. evaluated factor D levels and activity in healthy adults and in adults with advanced kidney disease, including 16 individuals on long-term dialysis and 20 with advanced kidney disease without dialysis. While renal replacement therapy may affect the findings in those with ESKD on dialysis, both groups with advanced kidney disease were found to have significantly higher levels of factor D than the healthy controls, and in the group not receiving dialysis, factor D levels correlated with the levels of creatinine (96). No significant differences in factor D levels were noted in individuals with nephrotic syndrome. In one patient with Fanconi syndrome, a significant increase in factor D levels in the urine was observed (96).

These data led Sanders et al. to conclude that factor D is ordinarily filtered by the kidney and reabsorbed by the proximal tubules, a conclusion further supported by microperfusion studies in the rat kidney (97). While the study by Volanakis et al. did not demonstrate increased levels of other biomarkers of the complement pathway, the functional hemolytic assay tended to increase in serum from patients with reduced kidney function and the authors postulated that faster kinetics of activation (i.e., increased tick-over) would ensue in advanced kidney disease (96). In another study, the same research group injected purified radiolabeled factor D into 5 healthy adults and 12 subjects with various degrees of kidney disease. They demonstrated that factor D synthesis was unaltered with reduced kidney function, but that clearance by the kidneys was reduced. As was noted in their prior study, factor D was increased in the urine of subjects with tubular dysfunction, indicating that it is ordinarily metabolized by the tubules. Importantly, this study confirmed that factor D levels increase in the circulation as kidney function declines (98). Several other studies examining the effects of factor D on alternative pathway activity have shown that in factor D–deficient serum, alternative pathway activity will increase in proportion to the concentration of factor D that is restored (99–101). Thus, supraphysiologic levels of factor D are sufficient to increase alternative pathway lability if other complement factors are held constant.

More recently, our group found that factor D levels were significantly increased in extracellular vesicles of 30 patients with stage 3 and 4 CKD (eGFR 20–59 mL/min/1.73 m^2^) as compared with healthy controls (102). There were no differences in factor H levels, but factor B levels were lower, consistent with alternative pathway activation and consumption of factor B. Notably, in an unbiased proteomics analysis, factor D was one of the most significantly increased proteins in the extracellular vesicles of the CKD subjects in comparison with healthy controls, with Ingenuity Pathway Analysis indicating a strong signal for complement, complement fragments, and complement regulator proteins (103). In contrast to the data by Volanakis et al., we found that soluble C5b-9, a biomarker of terminal complement activation, was significantly increased in the plasma of patients with CKD (102). We also found that factor D in the extracellular vesicles from the CKD subjects was functional and restored complement activity to factor D–depleted serum. This activity was prevented with coadministration of an inhibitory anti–factor D antibody.

In other data, we evaluated alternative pathway activation in 32 patients with stage 5 CKD not yet on dialysis secondary to C3G as compared with 33 patients with C3G but with normal kidney function (104). Factor D levels were significantly higher in those with stage 5 CKD, whereas factor H did not differ between groups. Additionally, in patients with factor D levels higher than 3 μg/mL, the higher levels of factor D associated with lower C3 and C5 levels for a given level of factor H, suggesting that the ratio of factor D to factor H is critical in inducing alternative pathway activation in the setting of reduced kidney function. To further understand the interplay between factor D and factor H, we evaluated complement regulation and C3 deposition in the kidneys of *Cfh*^–/–^;*Cfd*^–/–^ mice (104), a mouse model that develops a glomerular phenotype consistent with C3G. By administering human factor D and factor H intraperitoneally, we demonstrated that the concentration of factor D required to deplete the plasma complement was only 0.1 μg/kg, or only 0.1% of the factor D concentration in wild-type mice. In contrast, the minimum concentration of factor H required to control complement activity was approximately 30 μg/mL in the presence of a single dose of factor D (1 μg/kg, or 1% of total factor D in wild-type mice). These data indicate that even small increments in factor D can activate complement and significantly higher concentrations of factor H are needed to restore complement regulation in models predisposed to alternative pathway activation (104). Collectively, these data suggest that factor D increases with moderate-severe reduction in kidney function and that this increase may render the alternative pathway more labile in CKD.

### Factor H and FHR1

Factor H, the key inhibitor of the alternative pathway of complement, is a 155 kDa glycoprotein produced by the liver and composed of 20 repeating units called short consensus repeats (SCRs) (105). Factor H circulates in the plasma at high levels (180–420 μg/mL) (106). The complement inhibitory function of factor H is mediated by the four amino-terminal SCRs of the protein, while SCRs 6–8 and SCRs 19–20 facilitate binding to various ligands on cell and tissue membranes, thereby enabling complement regulation at these sites (105). Five FHRs (nos. 1–5) are also included in the factor H gene family and, like factor H, are modular proteins made of SCRs. Although shorter than factor H, the SCRs of the FHR proteins are structurally similar to SCRs 6–8 and 19–20 of factor H (107). As such, FHRs may antagonize the inhibitory effects of factor H on the alternative pathway at the tissue level (108). Consistent with this possibility, FHRs injected into murine models of kidney disease induce complement dysregulation in the kidney (105). In addition, homozygous deletion of *FHR1*, which is a common copy number variation, associates with lower risk of certain complement-mediated diseases including AMD, C3G, aHUS, and IgA nephropathy (109–111), while increased levels of FHR1 are seen in C3G and IgA nephropathy (63, 112). FHR1 and FHR5 are of particular interest as they promote C3b deposition to surfaces, which may allow for the continued activation of complement (113, 114).

FHR1 levels also appear to correlate with kidney function, as levels of FHR1 increase with reduced estimated GFR (63, 64). This association between increased levels of FHR1 and reduced kidney function is evident not only in IgA nephropathy but also in patients with CKD secondary to autosomal dominant polycystic kidney disease, a noncomplement-mediated kidney disease, suggesting that reduced kidney function increases FHR1 plasma levels (64). Considering that factor H levels do not change with CKD, the increased levels of FHR1 may alter FHR1/factor H ratios, which may be another mechanism by which CKD contributes to alternative pathway activation. In particular, a high FHR1/factor H ratio may contribute to complement activation in the extracellular matrix (115, 116). Additional metabolic alterations that are common in CKD, such as metabolic acidosis and ammonia production, may further disrupt the balance of activation and regulation (70, 117). It is therefore possible that CKD increases alternative pathway lability by several mechanisms.

### Complement fragment Ba and other complement biomarkers

Complement fragment Ba is a 33 kD cleavage product of factor B that is elevated in individuals with CKD (118). Oppermann et al. evaluated Ba levels (among other biomarkers of the alternative pathway) in 59 patients with advanced CKD without dialysis and in 61 patients with kidney failure receiving dialysis; 55 healthy subjects were included as controls. Ba levels were increased in those with CKD on dialysis (16.1 ± 6.1 μm/mL). While dialysis may confound these findings, it is notable that Ba levels were also increased in those with CKD not receiving dialysis (4.85 ± 3.58 μg/mL), albeit to a lesser extent than in those receiving dialysis (118). In addition to higher levels of Ba with CKD, Bb, activated C3, and factor D levels were noted to be significantly elevated. Consistent with data from other studies and as above, factor H levels were not different between groups. Another notable observation from this study was that Ba levels correlated significantly with factor B levels in those with CKD on dialysis, suggesting that the rate of factor B synthesis was an important determinant of Ba levels in this group of patients. Finally, levels of factor D and Ba normalized in 7 patients after kidney transplantation. The Ba fragment is small enough to filter through the functional kidney. While the increase in Ba levels may reflect reduced kidney clearance, higher levels of Bb (60 kD) provide evidence of activation of the alternative pathway in CKD.

In a more recent study, Yamane et al. measured inulin clearance and several biomarkers of the alternative pathway in 40 subjects with varying degrees of non–immune-mediated CKD, including stages 1–5 (119). They reported a significant correlation between Ba levels and inulin clearance but no such association was observed between inulin clearance and C5a or soluble C5b-9. Further scrutiny of these data indicates a trend toward higher levels of soluble C5b-9 with lower kidney function. The groups with stage 4 and 5 CKD were notably small (*n* = 6 and 1, respectively), and it is likely that the study was not adequately powered to achieve significance (119). Finally, changes in the levels of C3 and properdin could also affect alternative pathway activity. However, levels of these proteins do not seem to be directly affected by reductions in GFR (120, 121). These data, summarized in Table 1, suggest that increased levels of biomarkers of the alternative pathway reflect increased activity of this pathway in CKD.

## Systemic consequences of increased alternative pathway activity

Based on the existing literature, it is important to consider the potential role of alternative pathway activation in CKD-related complications. Future research should evaluate the degree of alternative pathway activation in CKD and the role this may play in CKD-related complications, including CVD and kidney disease progression. Importantly, identifying patients at the highest risk of alternative pathway–mediated injury is critical. We propose to consider the role of the alternative pathway in CKD in 2 stages, as follows:

### Stage 1: lability of the alternative pathway

As indicated by the evidence described above, when kidney function declines as in CKD, the levels of factor D, Ba, and FHR1 increase. The elevation of factor D and FHR1 render the complement system more labile. We propose that this increased lability is a universal phenomenon of CKD directly related to the degree of decline in kidney function.

### Stage 2: additional modifiers

We believe that some individuals with CKD are more susceptible to the development of complement activation based on CKD and non-CKD factors that further increase alternative pathway lability. These factors include the following:

#### Genetics.

This includes the complotype and whether it is at-risk or protective, the *CFH* haplotype (H1, H2, H3 and H4, which impact penetrance and phenotype of alternative pathway-mediated disease), and other aspects of their genetic background.

#### Baseline levels of complement regulators.

In healthy individuals, factor H levels vary from 180–420 mg/L. In two individuals with identical disease processes and identical CKD stages, therefore, the person with low normal levels of factor H will have higher complement lability at baseline and therefore greater disposition to CKD-associated complement activation than the person with high normal factor H levels. While both persons will suffer the complications associated with their CKD, the first person is more likely to additionally suffer complications from complement activation impacting various organ systems.

#### The underlying cause of their kidney disease.

If there is increased complement lability with the underlying kidney disease, as seen with C3G, aHUS, and IgA nephropathy, a progressive decline in kidney function and the associated increase in factor D and FHR1 would promote further complement activation leading to adverse outcomes.

#### The stage of CKD.

As many mediators of alternative pathway activation are inversely correlated with GFR, it is possible that the lability of the alternative pathway may play a larger role in more advanced stages of CKD. Alternatively, as kidney function declines, other CKD-related factors independent of the alternative pathway may be at play. This may be particularly relevant for those with ESKD receiving dialysis. As such, it is important to evaluate the potential impact of CKD stage on the degree of alternative pathway lability and/or activation and the contribution of alternative pathway activation to complications.

Several organs may be susceptible to increased alternative pathway activity, including the kidney and the eye. In particular, the glomerular basement membrane (GBM) in the kidney and Bruch’s membrane in the eye are considered specialized beds of extracellular matrix, typically not accessible to circulating complement proteins and lacking membrane-bound complement regulators such as CD55 and CD59 (122–124). However, in inflammatory settings, both sites may be exposed to circulating proteins and both have been shown to interact with components of the complement pathway (125, 126). Considering that factor H is the main complement regulator in these extracellular matrix beds, the GBM and Bruch’s membrane may be highly susceptible to increases in factor D and FHR1 as seen in CKD.

Alternative pathway dysregulation plays a critical role in the development and progression of several kidney diseases, including C3G, aHUS, postinfectious glomerulonephritis, IgA nephropathy, and antineutrophil cytoplasmic antibody–associated glomerulonephritis (127). In addition, the alternative pathway is known to play a role in AMD. Of note, several of the *CFH* polymorphisms that associate with C3G also associate with AMD onset and progression (81, 128–132). Of interest, beyond alternative pathway-mediated kidney diseases, several population level studies suggest that non–complement-mediated CKD, defined as reduced kidney function, significantly increases the risk of AMD (133–135). For example, a study by Weiner et al. showed that lower estimated GFR, but not albuminuria, increased the risk of late AMD with an odds ratio of 3.05 (95% confidence interval 1.5-6.1) independent of hypertension, diabetes, and body mass index (135). Based on the proposed paradigm, we postulate that the increase in the activity of the alternative pathway, which occurs in CKD, contributes, at least partially, to the high risk of progressive AMD in CKD.

Considering the constitutive activity of the alternative pathway and the continuous exposure of the endothelium to circulating complement proteins such as factor D and FHR1, the alternative pathway of complement has been postulated to play an important role in atherosclerosis (136). Cholesterol-containing lipid particles isolated from human atherosclerotic lesions have been shown to activate the alternative pathway in vitro and in vivo (137, 138). The generation of C3a and C5a further propagates vascular inflammation. In an animal model of heart transplantation, the alternative pathway was activated and contributed to ischemia-reperfusion injury while inhibitors of the alternative pathway reduced myocardial damage, cellular infiltration, and biomarkers of vascular inflammation including P-selectin, intercellular adhesion molecule-1, TNF-α, and IL-1β (139). Consistent with a potential role for the alternative pathway in ischemia-reperfusion injury, in humans undergoing percutaneous coronary procedures and diagnostic angiography, levels of the alternative pathway fragment C3bBbP were significantly higher in those with acute myocardial infarction on presentation versus those with stable angina, and C3BbP levels rose transiently during the procedure (140).

Whether CKD-induced lability of the alternative pathway contributes to the progression of atherosclerosis or the severity of myocardial injury during ischemia in CKD remains to be evaluated. Nevertheless, published literature supports multiple potential interactions between the alternative pathway and the vasculature. First, since the alternative pathway may contribute to progression of atherosclerosis (136), increased lability of the system in CKD could exacerbate disease. Acquired injury of the endothelium generates sites of alternative pathway activation (141), another phenomenon that may be exacerbated in CKD. Finally, many of the intracellular pathways induced by the alternative pathway intersect with those known to drive atherosclerosis, including activation of NF-κB signaling (142).

## Ongoing and future research

Based on evidence suggesting that CKD may contribute to alternative pathway lability, we are currently examining biomarkers and activity of the alternative pathway in CKD by a focused evaluation of samples from two clinical trials: Veterans Affairs Nephropathy in Diabetes (VA NEPHRON-D) (ClinicalTrials.gov NCT00555217) and the Systolic Blood Pressure Interventional Trial (SPRINT) (ClinicalTrials.gov NCT01206062). Further, our group is exploring whether the alternative pathway of complement mediates vascular dysfunction and inflammation and whether inhibitors of the alternative pathway improve vascular function in an animal model of CKD. Through a series of interrelated studies, we aim to define the mechanisms that underlie alternative pathway dysregulation in CKD, factors that may influence alternative pathway lability in CKD, and the potential impact of alternative pathway dysregulation on long-term kidney and cardiovascular outcomes. This understanding may define new therapeutic targets for this high-risk patient population.

**Figure 3:**
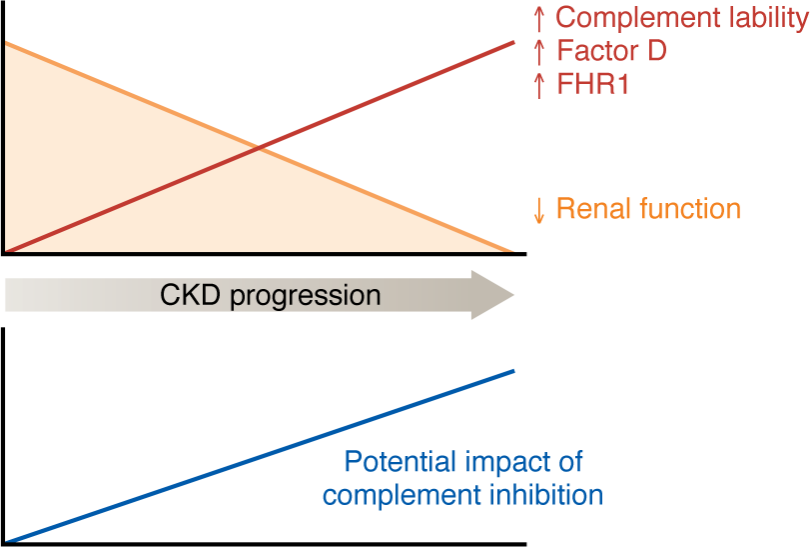
As kidney disease progresses, the lability of the alternative pathway increases. This may contribute to faster rates of progression of complement-mediated diseases such as deterioration of renal function in CKD. Lability of the alternative pathway may additionally contribute to CKD-associated complications. As complement lability increases, the potential beneficial impact of therapies that inhibit complement increases.

Multiple new anticomplement drugs are now available for clinical use and have been approved for several inflammatory diseases (143). Among the new drugs are agents that selectively block the alternative pathway, including drugs that specifically target factor D (144, 145). As illustrated in Figure 3, for patients with complement-mediated diseases, concomitant CKD may fuel alternative pathway activity and increase the severity of disease. This, in turn, may increase the benefit of complement inhibition. In the absence of an acute illness, alternative pathway inhibition may attenuate the adverse effects of CKD on CVD and kidney disease progression.

In summary, reduced kidney function is associated with increased levels of factor D in plasma. The increase in factor D may be sufficient to disrupt the balance of alternative pathway activation and regulation, which, in turn, may aggravate alternative pathway-mediated injury of the kidney and the cardiovascular system. Thus, the alternative pathway may link the loss of function in the kidney with a systemic inflammatory response and could eventually become a positive feedback loop. Alternative pathway inhibitors have recently been approved for use in specific kidney diseases but might eventually prove useful for preventing the systemic consequences of CKD.

## Figures and Tables

**Table 1:**
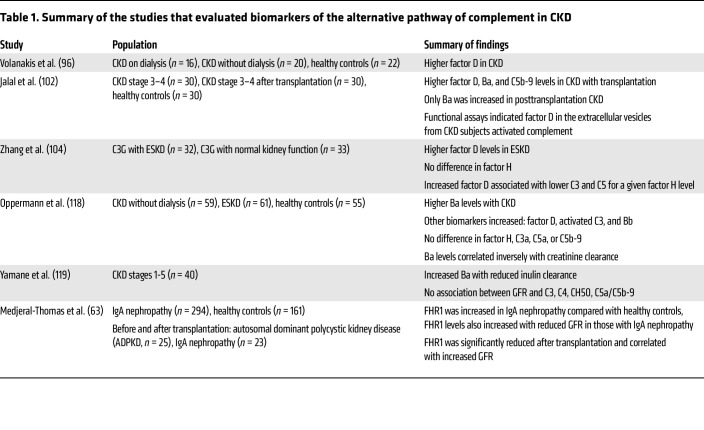
Summary of the studies that evaluated biomarkers of the alternative pathway of complement in CKD

## Version 1 05/01/2025

Electronic publication
